# Laryngitis

**Published:** 1829-03

**Authors:** 


### LARYNGITIS.

*Two Cases of Laryngitis, in which Bronchotomy was performed with success, at the* GLASGOW ROYAL INFIRMARY.

CASE I.—A weaver, fifty years of age, was admitted on the 7th of November, 1827, with dyspnœa and difficult deglutition. The voice was much impaired; the air, during inspiration, produced in its passage through the upper part of the larynx, a loud, moaning noise, and at times a ringing sound; occasional paroxysms of violent cough, with copious but difficult expectoration of tough and yellowish sputum; part in front, and at the side of the thyroid cartilage, swollen, and tender upon pressure, the swelling extending in a less degree towards the cricoid cartilage and os hyoides; no discoloration of the skin; nothing unusual in the fauces or the epiglottis; pulse 120, feeble, and thready; skin cold; aspect pale and haggard; strength much reduced.

These were the symptoms; and it seemed that, six weeks before, without obvious cause, the complaint began by swelling around the thyroid cartilage, followed by throbbing pain in the part. In the course of seven days the pain was relieved, but difficulty of breathing and swallowing commenced, and during the last eight days had been urgent.

Leeches, and after them a blister, were immediately applied over the larynx, and a grain of calomel and the same amount of opium ordered to be taken every third hour.

At nine P.M. he was suddenly seized with dyspnœa so severe as to threaten immediate suffocation; and this was still so urgent when Dr. Couper arrived, that he forthwith proceeded to open the windpipe. On account of the swelling above the larynx, the opening was made below the cricoid cartilage, which procured instantaneous relief for the dyspnœa. The wound was kept open by a bit of curved wire. No further difficulty of breathing took place, excepting that once a severe fit of coughing was caused by a little milk escaping through the wound. On another occasion, also, accidental derangement of the wire produced a slight paroxysm; but, further than these, not the slightest inconvenience was felt after the operation. After a few weeks the wire was exchanged for a curved silver tube, about two and a half inches long, and one fourth of an inch in diameter, provided with two small rings, through which a piece of tape was passed, and tied round the neck, to retain the tube in situ.

"On the supposition that the contraction of the cavity of the larynx depended on thickening of its lining membrane, a mercurial course was prescribed, but apparently without benefit; for, although the patient continued to breathe easily so long as the wound was kept open, yet all attempts to make him breathe through the mouth alone proved ineffectual. On various occasions the wound was closed with adhesive plaster, to ascertain if any improvement had taken place, but it was invariably found necessary, at the end of a few minutes, to open the wound, and replace the tube, on account of increasing dyspnœa. At one time I entertained hopes of being able to dilate the contracted larynx, by bougies passed upwards through it from the wound; but the extreme irritability of the parts rendered this proposal impracticable. The introduction of even a probe through the wound into the larynx was found to excite such a paroxysm of coughing, that it was absolutely necessary to desist."

After remaining in the hospital above five months, the patient was dismissed, suffering no inconvenience except the necessity of breathing through the tube, a circumstance which habit had rendered very tolerable. By stopping up the tube with the point of his finger, he could speak in a hoarse but audible tone. In the month of August last he appeared at the Infirmary, and was perfectly free from all complaint.

Case II.—This was a tobacco-pipe maker, aged twenty-eight, who was seen by Dr. Couper thirteen days after the commencement of the laryngeal inflammation. He had been bled pretty freely at different times, blistered, and treated with diaphoretic medicines; but still, though at times apparently relieved, the disease had proceeded on its march. When examined by Dr. C. he was just recovering from a fit of alarming orthopnœa, and presented these symptoms: incapability of assuming the horizontal posture; inspiration laborious and wheezing; fauces red and swollen; epiglottis enlarged, tense, and shaped like a glans penis during erection; uneasiness decidedly referred to the larynx.

"Laryngotomy was immediately agreed upon. In making the incision through the integuments, a small artery was cut, and bled very freely. At the same instant the dyspnœa became greatly increased; the patient's face became livid, his limbs quivered, and his urine was ejected involuntarily. Without waiting to secure the artery, I immediately perforated the thyro-cricoid membrane, and the transition from the state now described to easy respiration was nearly instantaneous. The patient's body being inclined forward, no inconvenience was felt from the bleeding, which was speedily stopped by the pressure of the wire employed to dilate the aperture. From this time he continued to breathe easily, partly by the wound and partly by the mouth, and swallowed without difficulty."

Four days after the operation the wire was withdrawn, and on the 15th the wound was so very nearly healed that even during coughing no air escaped by it. Nine days after this the patient had a rigor, followed by urgent orthopnœa, and a little pain and swelling of the right side of the larynx. After vainly employing a full dose of laudanum and antimonial wine, without relief, the larynx was opened a second time. In the course of ten days the wire was changed for a silver tube, which was kept in the wound for upwards of a month, and then withdrawn; shortly after which the wound was healed.

A few days after this he was discharged, affected with only a glandular swelling on the left side of the neck, which soon disappeared on his leaving the hospital.

"Both of these cases appear important: the former as an example of contraction of the larynx produced by chronic inflammation, and the latter as an instance of the same effect arising from acute œdematous laryngitis. The important fact that the dyspnœa, in cases of laryngeal disease, is liable to sudden and dangerous exacerbations, is well illustrated by both. Such paroxysms may cease after the irritability of the parts is exhausted, but they will certainly recur again and again, until suffocation is produced, unless an artificial opening is made into the windpipe, to allow a free access of air to the lungs. When the necessity for it ceases, the aperture can be easily healed up; and, even should the contraction of the larynx prove permanent, as in the case of Limpitlaw, it must be allowed that the inconvenience arising from breathing through a tube inserted into the windpipe, during the remainder of life, is small when compared with loss of a limb, to which few refuse to submit as a mean of prolonging life."

